# The application of the mobile application for the assessment of cleaning workers’ exposure to cleaning products: a pilot study

**DOI:** 10.1093/annweh/wxad082

**Published:** 2023-12-24

**Authors:** Sewon Lee, Andrew Povey, Martie van Tongeren

**Affiliations:** Centre for Occupational and Environmental Health, School of Health Sciences, University of Manchester, Manchester M13 9PL, United Kingdom; Centre for Occupational and Environmental Health, School of Health Sciences, University of Manchester, Manchester M13 9PL, United Kingdom; Centre for Occupational and Environmental Health, School of Health Sciences, University of Manchester, Manchester M13 9PL, United Kingdom

**Keywords:** barcode, cleaning product, respiratory health, smartphone

## Abstract

**Background:**

Cleaning product use has been associated with adverse respiratory health effects such as asthma in cleaning staff and healthcare workers. Research in health effects from cleaning products has largely depended upon collecting exposure information by questionnaires which has limitations such as recall bias and underestimation of exposure. The aim of this study was to develop a Cleaning and Hazardous Products Exposure Logging (CHaPEL) app with a barcode scanner and to test the feasibility of this app with university cleaners.

**Methods:**

The CHaPEL app was developed to collect information on demographics, individual product information, and exposure information. It also included an ease-of-use survey. A pilot study with university cleaning workers was undertaken in which cleaning workers scanned each product after use and answered the survey. Respiratory hazards of cleaning substances in the scanned cleaning products were screened by safety data sheets, a Quantitative Structure-Activity Relationship model and an asthmagen list established by an expert group in the US.

**Results:**

Eighteen university cleaners participated in this study over a period of 5 weeks. In total, 77 survey responses and 6 cleaning products were collected and all reported that using the app was easy. The most frequently used product was a multi-surface cleaner followed by a disinfectant. Out of 14 substances in cleaning products, ethanolamine and Alkyl (C12-16) dimethyl benzyl ammonium chloride were found as respiratory hazardous substances.

**Conclusion:**

The CHaPEL app is a user-friendly immediate way to successfully collect exposure information using the barcodes of cleaning products. This tool could be useful for future epidemiological studies focused on exposure assessment with less interruption to the workers.

**What’s Important About This Paper?**

The Cleaning and Hazardous Products Exposure Logging (CHaPEL) app is a novel smartphone application that provides a user-friendly method for immediate exposure information collection by scanning product barcodes and administering simple questionnaires. It has the potential to improve data quality by reducing recall bias and minimizing interruptions for participants, thus overcoming limitations commonly associated with traditional questionnaires. With the inclusion of health surveys, this tool could prove invaluable for further large-scale epidemiological studies.

## Introduction

Cleaning product use has been associated with adverse respiratory health effects such as asthma in cleaning staff (Medina-Ramón et al. 2003; Vizcaya et al. 2011) and healthcare workers (Delclos et al. 2007; Gonzalez et al. 2014). Exposure to cleaning products in epidemiological studies has generally been assessed based on the types of cleaning products (Zock et al. 2007; Le Moual et al. 2012) or cleaning tasks (Obadia et al. 2009), not based on respiratory hazardous substances in cleaning products.

Furthermore, exposure to cleaning agents has been assessed by self-reporting (Vizcaya et al. 2011, 2015; Le Moual et al. 2012), expert assessment (Donnay et al. 2011), and job-exposure matrices (Goldberg et al. 1993; Dumas et al. 2012) based on a questionnaire. Data collected by questionnaire relies on the participants’ memory, which means it is subject to recall bias (Siracusa et al. 2013).

Smartphone applications with a barcode scanner can be used to collect exposure information conveniently and improve data collection for identifying substances. The smartphone app is expected to be more advantageous for reliably and efficiently obtaining information about substances in household products in a large population study (Elena et al. 2018). A recent study showed fair agreement between information about the weekly use of a few household cleaning products collected from questionnaires and smartphones (Lemire et al. 2021).

However, many cleaners are low-paid, non-English speakers and have a low level of education. Hence, there is concern that cleaners may not be able to engage appropriately with a smartphone application to provide reliable data. Therefore, we aimed to develop and test the Cleaning and Hazardous Products Exposure Logging (CHaPEL) application to help collect information on substances in cleaning products and exposure to these substances. Moreover, respiratory health hazards of product constituents were identified by three different methods.

## Methods

### Cleaning and Hazardous Products Exposure Logging (CHaPEL) App

The CHaPEL app has been developed based on C# language by Research IT team at the University of Manchester. The CHaPEL app consists of four parts. All questions except the barcode scanning were multiple choices, with checkboxes laid out in a user-friendly way (Supplementary Files 1 and 2).

1. Demographic survey: participant’s age, gender, and first language (required only once at first use).
2. Barcode scanning: barcode scanner and barcode typing bar.
3. Product use survey: dilutions, places used, ventilation status, time for use, and amount used.
4. Ease-of-use survey: how easy it was to use this app, the difficulty of the survey, and how much this app use interrupted the user’s tasks (not mandatory, required only once at first use).

A participant user scanned or typed the barcode of the product, which links it with the database that contains information on the cleaning products, after finishing his/her work shift. Afterwards, the participant answered the product use survey. The scanned or typed product barcode was recognized and matched with the barcode in the database. An individual participant was automatically provided with a unique anonymous ID, and answers were automatically sent to the University’s Central Authentication Service platform, StorageConnect, when connected to wi-fi.

## The database of cleaning and disinfection products

The CHaPEL app is linked to a database of 580 cleaning products, including 571 products used in NHS organizations across England and Wales, and 9 products regularly used at the University of Manchester (UK) (6 products initially added before the study and 3 additional products updated during the study). Out of 230 substances, sodium carbonate was the most frequently found substance followed by isopropanol in 52 products. Product information was added to the database such as barcode, product name, form, substances, and Safety Data Sheet (SDS) link.

The SDS was used to identify substances in each product and their respiratory hazards, based on H-statements (e.g. H334 - respiratory sensitization, H335 - respiratory irritation). Furthermore, two additional methods were used to identify potential respiratory hazards (Lee et al. 2021).

- a QSAR model to identify low-molecular organic compounds (<1,000 Da) that potentially have respiratory sensitization characteristics (Jarvis et al. 2005).
- List of asthmagens developed by the Assocation of Occupational and Environmental Clinics (AOEC) in the US based on the criteria to cause sensitizer/irritant-induced occupational asthma (Rosenman and Beckett 2015).

Details of the cleaning product database are presented elsewhere (Lee et al., unpublished).

### Testing the CHaPEL and descriptive analysis

Eighteen cleaners signed the consent form to volunteer to participate for three to five days a week from 8 September to 8 October 2021. Each individual participant was provided with a smart phone with the CHaPEL application pre-downloaded. The ethics approval for this study was obtained on 19 November 2020 from the University of Manchester Proportionate UREC process (Ref: 2020-9014-17018). All data were imported and analysed descriptively using R.

## Results

### The total number of participants and data for analysis

Eighteen cleaners (11 males and 7 females) participated in the test between 3 and 5 days weekly, depending on their workload. In total, 7 cleaning products and 118 product use survey records were collected in 48 person-day. (1 to 4 days by each participant). A total of 106 (90%) were correctly linked to the product database. Among 12 records not found in the database, 8 barcodes were 1 or 2 digits different from the barcodes of registered products. 1 barcode was non-cleaning product’s barcode (soap) and the 3 barcodes were not identified. Afterwards, 37 further records were excluded since survey inputs with the same product were submitted more than once a day. At last, 77 records (65.3%) of data linked with the database were used as the final data for the following descriptive analysis.

### Descriptive analysis of the data collected by a mobile app

**Fig. 1.**
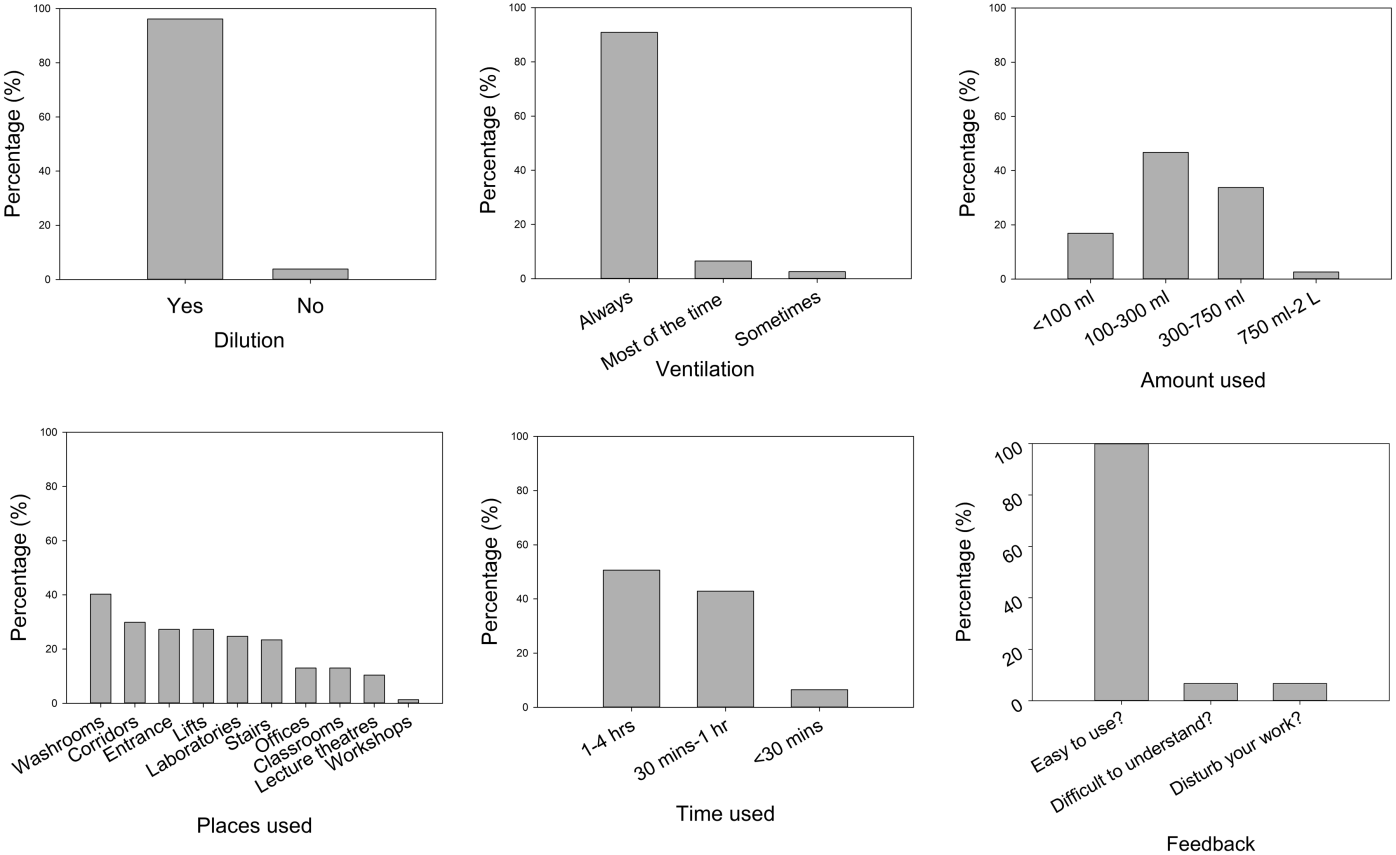
Descriptive exposure survey results (Dilution, Ventilation, Time used, Amount used, Places used, Feedback).

The majority of participants (*n* = 15) were aged over 40. All participants’ first language was English. A total of 6 products were used, and their form was liquid/emulsion. On each day, one participant used 1.8 ± 0.6 (1 to 4) products. Most participants reported that they diluted the cleaning products before use in well-ventilated areas and that products were used in well-ventilated rooms. Cleaning products were generally used for long periods (50% >1 h). In 80% of cases, the amounts of product used were 100–750 ml—between less than 1/3 and 1 spray trigger bottle. Washrooms were the places where a product was used most frequently: 40.3% (Fig. 1). All cleaners (*n* = 15) reported that using the app was easy. Only 1 participant answered that it was difficult to understand the questionnaire, and participant reported their work was interrupted due to the use of the app.

Fourteen substances were found in at least one product among the 6 products that the cleaners used, of which 2 were respiratory hazards (Table 1). Alkyl (C12-16) dimethyl benzyl ammonium chloride is included in cleaning products that were used during most days (42 person-day) and was listed as an asthmagen in the AOEC list. Ethanolamine was also frequently used (27 person-day) and is recognized as a respiratory irritant based (H335) and as an asthmagen and potential respiratory sensitizer by the AOEC list and QSAR model, respectively.

**Table 1.** Substances in cleaning products and their hazards.

Frequency (person-day)	Substances	CAS RN	SDS	AOEC	QSAR
44	Alcohol (c9-11) ethoxylate (8eo)	68439-46-3	H302	N	N/A
42	C12-15 alcohol ethoxylate (7eo)	68131-39-5	H302, H318	N	N/A
**42**	**Alkyl (c12-16) dimethyl benzyl ammonium chloride**	**68424-85-1**	**H302, H314, H318**	**Rs** ^*^	**N/A**
**27**	**Ethanolamine**	**141-43-5**	**H302, H312, H314, H318, H332, H335**	**Rs** ^*^	**Rs** ^*^
26	Amino trimethylene phosphonic acid penta sodium salt	2235-43-0	H315, H318	N	N
26	Cocamidopropyl betaine	61789-40-0	H318	N	N
8	2- Butoxyethanol	111-76-2	H302, H312, H315, H319, H332	N	N
7	Propan-2-ol	67-63-0	H319, H336	N	N
1	2-(2-Butoxyethoxy)ethanol	112-34-5	H319	N	N
1	Sodium hydroxide	1310-73-2	H314	N	N/A
1	1,2-benzisothiazolin-3-one	2634-33-5	H302, H315, H317, H318	N	N
1	Sodium cumene sulphonate	32073-22-6	H319	N	N
1	Sodium metasilicate	6834-92-0	H314, H318	N	N/A
1	Sodium dodecyl benzene sulphonate	68411-30-3	H302, H315, H318	N	N/A

**In bold: Respiratory hazardous substances**

^*^Rs: Respiratory sensitizer, N/A: Not applicable

H302: Harmful if swallowed, H312: Harmful in contact with skin, H314: Causes severe skin burns and eye damage, H315: Causes skin irritation, H317: May cause an allergic skin reaction, H318: Causes serious eye damage, H319: Causes serious eye irritation, H332: Harmful if inhaled, H335: May cause respiratory irritation

## Discussion

The CHaPEL app was developed to collect information from cleaners and healthcare workers on exposure to cleaning products. Most submitted barcodes were identifiable by the database. Survey answers for collecting exposure information from cleaners were successfully transferred and saved. The participants responded that the CHaPEL app was easy to use and did not generally interrupt their work. Out of 14 substances contained in the 6 cleaning products they used, ethanolamine and Alkyl (C12-16) dimethyl benzyl ammonium chloride were identified as respiratory hazardous substances by at least one method. However, the SDS does not include all components, i.e. chemicals below 1.0 or 0.1% are generally not included in the SDS (Gerster et al. 2014). Furthermore, the QSAR model used in this paper can only be used for low-molecular-weight (<1,000 Da) organic compounds (Jarvis et al. 2005).

The survey questions in CHaPEL were relatively simple and basic questions and did not include any questions on health symptoms. Also, 3 mistyped barcodes, 1 non-cleaning product’s barcode, and 37 multiple records on the same product in the same day were observed in this study. It might be challenging to use this app for other studies with larger populations and many products. Nevertheless, the CHaPEL app has advantages. Firstly, the app can be downloaded publicly and works on both Android and iOS. Since inputs are transferred to the server directly, the inputs could be stored safely and nearly permanently and downloaded instantly at any time.

Smartphone applications with barcode scanner could be used to improve data collection on exposure to cleaning products. In one study (Bennett et al. 2012), the barcode scanner was used to obtain information such as the type of household cleaning and personal care products, and the amount of product used. Also, good information on healthcare worker’s exposure to 50 different cleaning products—such as frequency of use and type of products—was obtained by a smartphone application (Quinot et al. 2018). However, the app in the other study was not able to operate on iOS, and the study was conducted under the researcher’s observation (Quinot et al. 2018)

## Conclusion

The CHaPEL app is a user-friendly way to collect exposure information immediately by scanning barcodes of products and completing a simple questionnaire. Therefore, this CHaPEL application could help further exposure or epidemiology studies with less interruption to the workers.

## Supplementary Material

wxad082_suppl_Supplementary_Files

## Data Availability

The data underlying this article will be shared on reasonable request to the corresponding author.
