# Supplementary material for: The application of the mobile application for the assessment of cleaning workers’ exposure to cleaning products: a pilot study
Source: Ann Work Expo Health. 2023 Dec 24;68(2):211–6. doi: 10.1093/annweh/wxad082 (PMC10877463; doi:10.1093/annweh/wxad082)
Supplement: wxad082_suppl_Supplementary_Files [file wxad082_suppl_supplementary_files.docx]

# The application of the mobile application for the assessment of cleaning workers' exposure to cleaning products: a pilot study

Authors

Sewon Lee^1^, Andrew Povey^1^, Martie van Tongeren^1*^

Author affiliations

1. Centre for Occupational and Environmental Health, School of Health Sciences, University of Manchester, Manchester M13 9PL, UK

Correspondence to Professor Martie van Tongeren, Centre for Occupational and Environmental Health, 4th Floor, Ellen Wilkinson Building, Oxford Road, Manchester M13 9PL, UK. Tel: +44 (0); fax: +44 (0); e-mail: Martie.J.Van-Tongeren@manchester.ac.uk


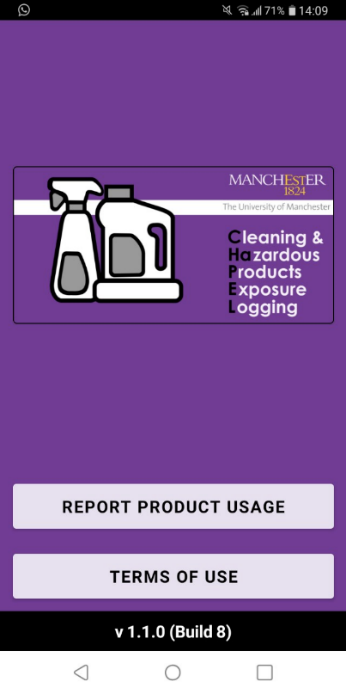

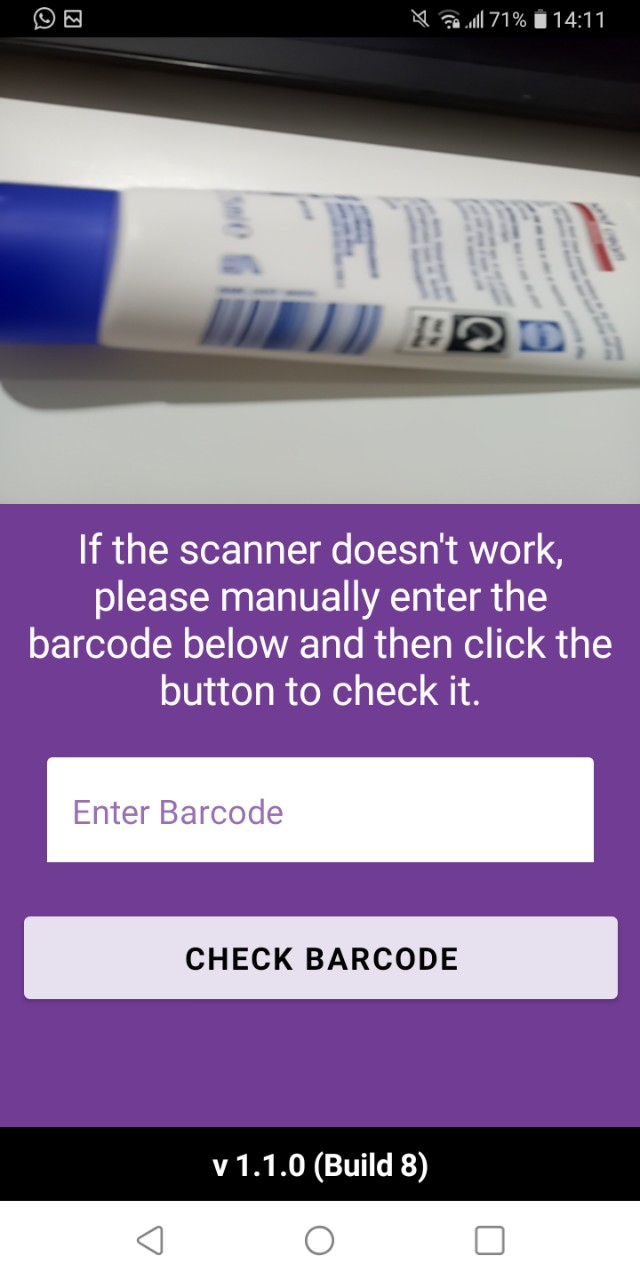


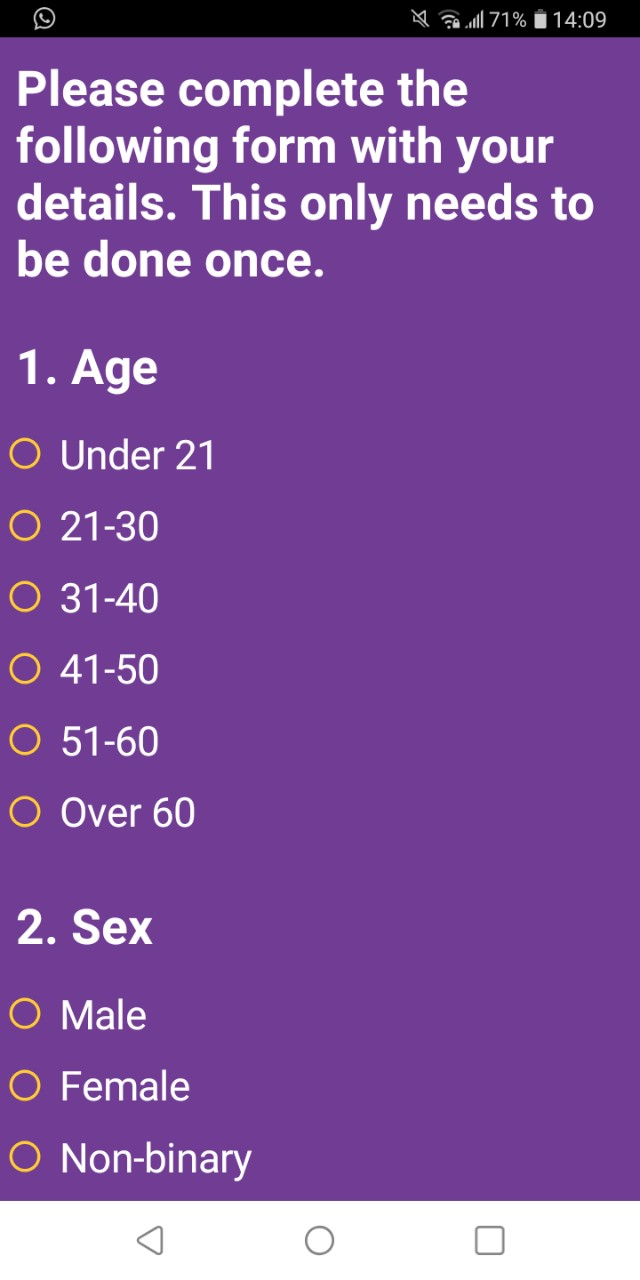

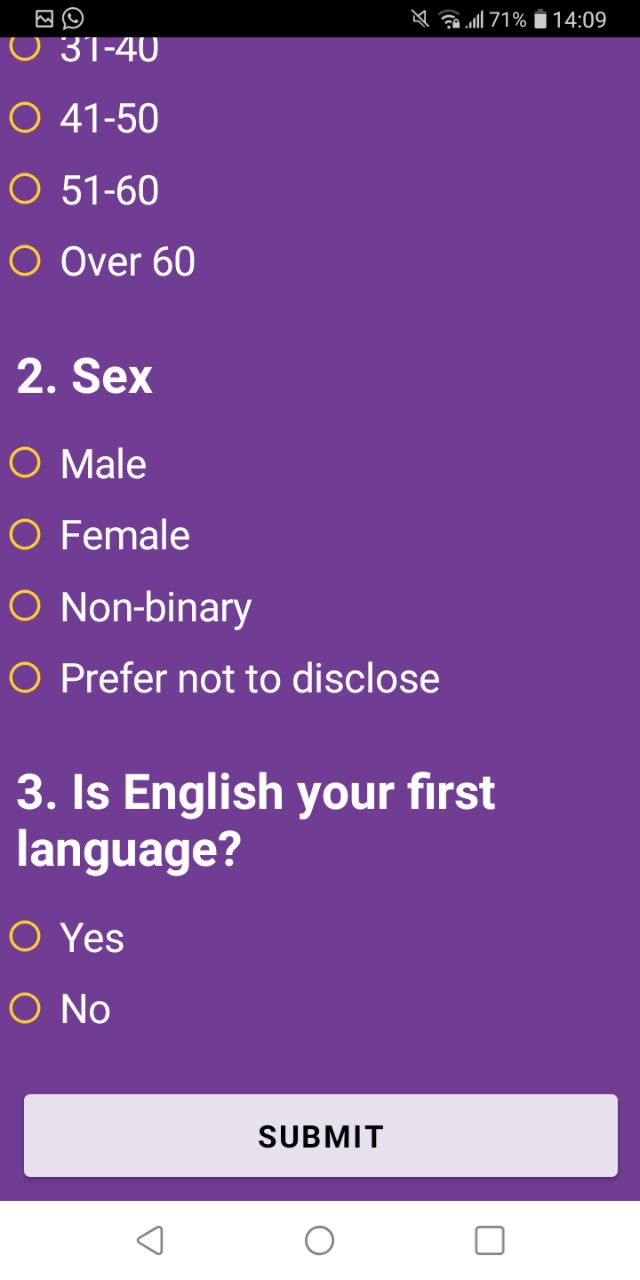


a. First screen b. Demographic survey c.Barcode scanner


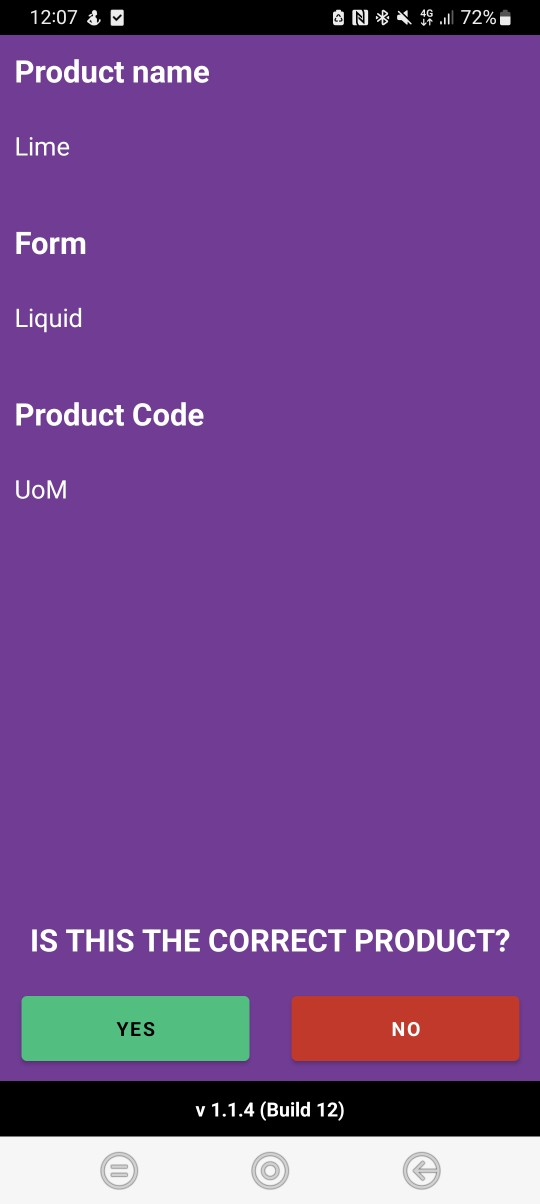

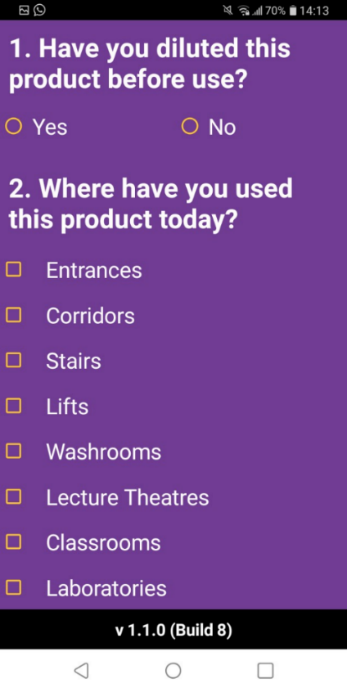

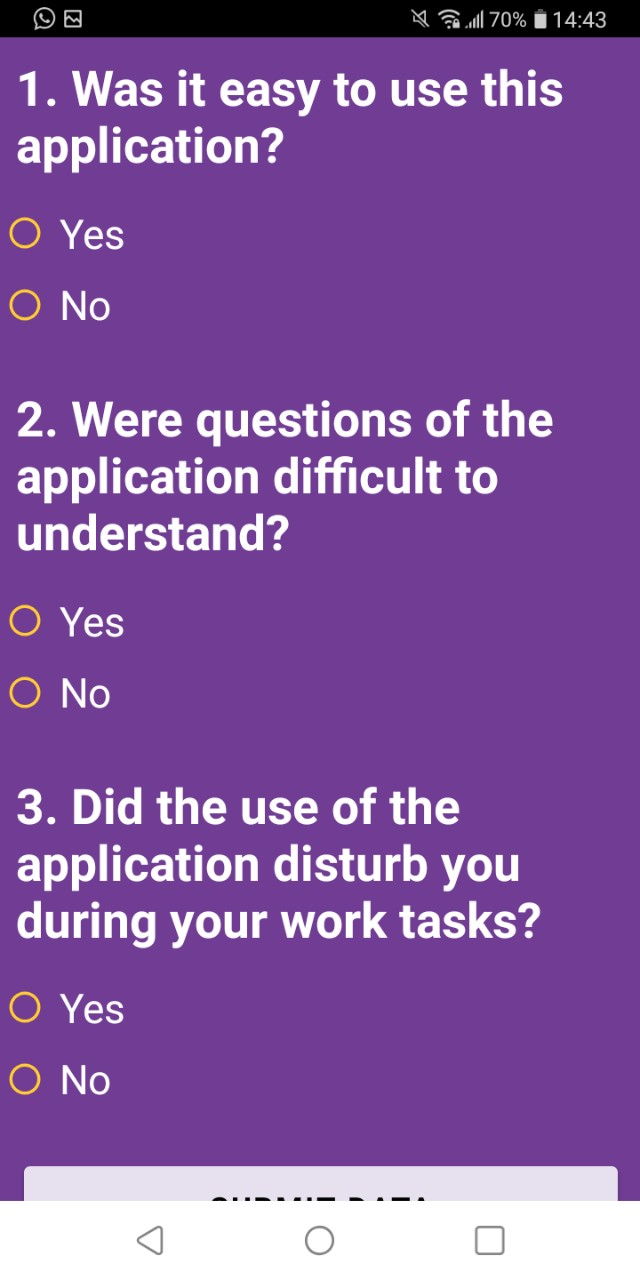


d. Successfully scanned product e. Product use survey f. Ease of use of the

information CHaPEL app

# Supplementary 1 Screenshots of the CHaPEL application

**Demographic survey**

1. Age

□ <21 □ 21-30 □ 31-40 □ 41-50 □ 51-60 □ >60

1. Gender

□Male □Female □Non-binary □Prefer not to disclose

1. Is English your first language?

□Yes □No

**Exposure survey**

1. Have you diluted this product today before use? (if you checked ‘No’, go to Question 2)

□ Y □ N

- 1. Was the area in which you diluted it well-ventilated? (i,e. by opening window/door/extractor hood)

□ Yes □ No

1. Where have you used this product today? (Multiple choices available)

□ Entrance □ Corridor □ Stairs □ Lifts□ Offices □ Washrooms □ Lecture Theatres □ Classrooms □ Laboratories □ Workshops

1. Do you consider that these areas where you worked today were well-ventilated?

□ Yes, I am always able to open windows

□ Most of the time, but I am not always able to open windows

□ Sometimes, but usually there is no option to open windows

□ Never

1. How long have you used this product in total today approximately?

□ <30mins □ 30mins-1hr □ 1-4hrs □ >4hrs

1. How much of this product have you used in total today approximately?

□ <500ml □ 500ml-2L □ 2-5L □ >5L

**Feedback (only once)**

1. Was it easy to use this application?

□ Y □ N

1. Were questions of the application difficult to understand?

□ Y □ N

1. Did the use of the application disturb you during your work tasks?

□ Y □ N

# Supplementary 2 All survey questionnaire on the CHaPEL app
